# A brain-region-based meta-analysis method utilizing the Apriori algorithm

**DOI:** 10.1186/s12868-016-0257-8

**Published:** 2016-05-18

**Authors:** Zhendong Niu, Yaoxin Nie, Qian Zhou, Linlin Zhu, Jieyao Wei

**Affiliations:** School of Computer Science, Beijing Institute of Technology, Beijing, China; Beijing Engineering Research Center of High Volume Language Information Processing and Cloud Computing Applications, School of Computer Science, Beijing Institute of Technology, Beijing, China; The Information School, University of Pittsburgh, Pittsburgh, PA 15260 USA

**Keywords:** Apriori algorithm, Brain network connectivity, Co-activation relationship, fMRI, Meta-analysis, Word reading

## Abstract

**Background:**

Brain network connectivity modeling is a crucial method for studying the brain’s cognitive functions. Meta-analyses can unearth reliable results from individual studies. Meta-analytic connectivity modeling is a connectivity analysis method based on regions of interest (ROIs) which showed that meta-analyses could be used to discover brain network connectivity.

**Results:**

In this paper, we propose a new meta-analysis method that can be used to find network connectivity models based on the Apriori algorithm, which has the potential to derive brain network connectivity models from activation information in the literature, without requiring ROIs. This method first extracts activation information from experimental studies that use cognitive tasks of the same category, and then maps the activation information to corresponding brain areas by using the automatic anatomical label atlas, after which the activation rate of these brain areas is calculated. Finally, using these brain areas, a potential brain network connectivity model is calculated based on the Apriori algorithm. The present study used this method to conduct a mining analysis on the citations in a language review article by Price (Neuroimage 62(2):816–847, 2012). The results showed that the obtained network connectivity model was consistent with that reported by Price.

**Conclusions:**

The proposed method is helpful to find brain network connectivity by mining the co-activation relationships among brain regions. Furthermore, results of the co-activation relationship analysis can be used as a priori knowledge for the corresponding dynamic causal modeling analysis, possibly achieving a significant dimension-reducing effect, thus increasing the efficiency of the dynamic causal modeling analysis.

## Background

Functional neuroimaging, especially functional magnetic resonance imaging (fMRI), is an indispensable method for non-invasively researching human brain function. fMRI is not only used to study the function of a particular brain region, but is also increasingly being used to establish the network structure of the brain. The human brain is comprised of highly complex networks with hundreds of billions of neurons and more than 100 brain regions [1]. The various brain regions work both independently and collaboratively to complete certain cognitive tasks. Over the last 20 years, many studies have investigated brain activation using fMRI. However, most of these studies only examined brain activation in response to a specific task; while we gained knowledge of discrete brain areas by those studies, we still lack information about the functional integration (connections) among them.

Meta-analysis is an increasingly popular and valuable tool for summarizing results across many neuroimaging studies. Currently, two meta-analysis methods are popular in the brain imaging literature: the activation likelihood estimation (ALE) meta-analysis method [2, 3] and the meta-analytic connectivity modeling (MACM) method [4]. The ALE meta-analysis method can effectively integrate studies with consistent results by using statistical analyses; however, ALE is unsuitable for modeling the existing functional connections in the brain. On the other hand, the MACM method is based on the BrainMap database [5], which examines the relationships and connections between a particular region of interest (ROI) and other ROIs. Furthermore, MACM can overlay the results of separate analyses of multiple ROIs to obtain the corresponding network connectivity model.

In this paper, we present a new meta-analysis method for mining the co-activation relationship of brain regions without using ROIs. Our method focuses on the functional brain connectivity under the same task. This method uses the automatic anatomical label (AAL) atlas to define the brain region of each foci reported, and then applies the Apriori algorithm [6] to calculate the co-activation relationships. To confirm the effectiveness of this method, we employed part of a literature review [7] containing a meta-analytic dataset and compared the results of our meta-analysis to those obtained in the literature review. Furthermore, the possible dimension-reducing effects of this method on the corresponding dynamic casual modeling (DCM) analysis were analyzed. This will enable a higher efficiency in DCM when analyzing the effective connectivity of multiple ROIs.

## Methods

The proposed method aimed to find the co-activation relationships among brain regions from a dataset consisting of neuroimaging studies. The method includes three steps. First, all activation foci are assigned to the identified brain regions. Second, brain regions that frequently appear across the studies are identified using an association analysis. Finally, the associated network of related brain regions is calculated using the Apriori algorithm. The details of the new method are described below.

Our brain region activation probability model is based on the voxel activation probability model of the ALE method [2]. When modeling the voxel activation, we assumed that for a certain voxel coordinate X_i_, the probability of being activated at the peak point (x, y, z) of a certain region is:

1 $$\Pr (X_{i} ,a) = \frac{{e^{{( - d_{i}^{2} /2\sigma^{2} )}} }}{{(2\pi )^{1.5} \sigma^{3} }}$$

where d_i_ is the Euclidean distance from X_i_ to point (x, y, z), and σ is the standard deviation of the distribution.

For a point in the brain X_i_, the overall probably that this point will be activated can be calculated as follows:

2 $$\begin{aligned}\Pr (X_{i} ,a,b) &= \Pr (X_{i} ,a) + \Pr (X_{i} ,b)\\ &\quad - \Pr (X_{i} ,a)*\Pr (X_{i} ,b) \end{aligned}$$

where a, b indicates a different peak coordinate for activation (note that if there are more than two peak coordinates, then formula (2) will change according to the inclusion–exclusion principle in probability).

Formula (2) can be used to obtain the probability of each point in the brain being activated in each study and the probability distribution, which are then represented as a brain activation probability distribution diagram. Then we can obtain the border of a brain region’s anatomy, V, by querying the anatomical boundaries found in various brain region annotated databases. Here, we used the AAL atlas. An integral was used to determine the boundary of the activated region. For a particular brain region, the possibility that it is active is defined as:

3 $$\Pr (V) = \iiint\limits_{V} {\Pr (X_{i} )dv}$$

where, Pr (X_i_) is the probability that point X_i_ is activated in region V, which is obtained in the first step, and Pr(V) is the probably that a certain region V is activated. The region is output by name when Pr(V) is greater than a certain threshold t, thus obtaining the activated anatomical region in the study. The finer the division of a brain region’s anatomical boundaries, the more accurate the calculation of the brain network.

The Apriori algorithm [6] mainly uses prior knowledge of the data to perform its analyses; therefore, it can take advantage of mining the frequent itemsets. Here, we adopted the A-Close algorithm [8] of the Apriori algorithm.

The A-Close algorithm also mainly uses prior knowledge, as follows: (1) a subset of all frequent itemsets is also a frequent itemset, (2) a superset of all of the non-frequent itemsets is also non-frequent, (3) the degree of support of a frequent itemset is equal to its closure, and (4) frequent itemsets only have one closure.

Here, we made the following provision: A is an exhaustive list of brain activation regions under a particular cognitive state; support (A) indicates the number of times that list A appears in several articles divided by the total number of articles. If support (A) is greater than a threshold value, such as 0.8, then it is termed a frequent itemset, and the threshold value is named a degree of support of A. The possibility of determining connections between brain regions from a particular brain origin is a very large inner frequent itemset. In a frequent itemset A, the confidence coefficient for deducing region b from brain region a is calculated as:

4 $$confidence \, \left( {A \to B} \right) = \frac{{{\text{support }}(A \cup B)}}{{{\text{support }}(A)}}$$

It is assumed that there is a connection from A to B if confidence (A → B) is greater than a threshold value, such as 0.7.

This method first identifies the sets of brain regions that are activated frequently in studies utilizing similar tasks. Then, it obtains the degree of mutual influence within each brain area by calculating the confidence coefficient among the brain areas in this set. Finally, it generates a co-activation relationship model of the brain for studies utilizing the same tasks.

To demonstrate our methods, we used the reference list within a recent review article [7] as our dataset; the review article we used discussed the brain areas associated with heard speech, speech production, and reading. The benefits of utilizing such experimental materials are two-fold. First, all of the studies used the same type of experiment, that is, experiments activating brain processes reflecting orthography mapping to phonology. This ensured that the experimental data were not affected by noise interference, which improved the accuracy of the analysis results. Second, as the experimental materials were collected from an authoritative review paper, we can verify the correctness of the algorithm by comparing the network reported by Price [7] with the network obtained by the automatic analysis.

The paper by Price [7] is divided into several sections, and the final section, “Dissociating neural pathways for mapping orthography to phonology,” discusses how orthography is mapped to phonology when the brain performs reading tasks. The process is generally divided into two stages, namely orthographic analysis and orthographic coding mapped to phonology. The author, through the inductive analysis of the review in the above-mentioned section, concludes that there is a brain network for completing the above two stages.

There are 62 studies mentioned in the “Dissociating neural pathways for mapping orthography to phonology” section of the review. Thirty-three studies (Table 1) that fulfilled the following criteria were included in our meta-analysis: (1) used fMRI or positron emission tomography as the imaging modality, (2) applied visual tasks that required speech processing, (3) used whole-brain scanning and reported complete coordinates of the activation in standardized stereotaxic space [the Talairach atlas or the Montreal Neurological Institute (MNI) atlas], and (4) recruited healthy subjects.

**Table 1:** Experiments included in the meta-analysis

Study	N	Task and contrast	No. of foci
Binder et al. [9]	24	Regular word naming > non-word naming	32
Bolger et al. [10]	46	Mean activation for reading	27
Bolger et al. [11]	24	Normal orthographic and phonological judgment > Impaired	24
Cohen et al. [12]	12	Word degradation > baseline	16
Dietz et al. [13]	16	Reading > fixation	4
Edwards et al. [14]	18	Mean activity in all tasks	10
Fiebach et al. [15]	12	Word naming > pseudo word naming	14
Frost et al. [16]	22	Word naming > baseline	3
Graves et al. [17]	27	Forward words > reversed words	12
Hagoort et al. [18]	11	Silent word reading > fixation	7
Herbster et al. [19]	10	Irregular + regular words read aloud > zero-order speak	6
Hu et al. [20]	25	Normal semantic word matching > dyslexic semantic word matching	6
Ino et al. [21]	14	Word recognition > reading aloud	30
Lee et al. [22]	20	Main effect of word frequency in reading	26
Levy et al. [23]	16	Orthographic conjunction analysis	8
		Phonological conjunction analysis	6
Liu et al. [24]	16	Meaning judgment > line	12
		Rhyming judgment > line	12
Matsuo et al. [25]	33	Homophone judgment > baseline	18
McDermott et al. [26]	20	Phonologically related words > semantically related words	20
Mechelli et al. [27]	6	Word reading > rest	21
Mechelli et al. [28]	20	Reading words > fixation	10
Mechelli et al. [29]	22	Reading > false fonts	6
Meschyan and Hemandez [30]	12	Reading Spanish word silently > rest	12
Nosarti et al. [31]	30	Irregular words > regular words	11
Owen et al. [32]	8	Overt pseudohomophone naming > non-word rhyming decision	7
Price et al. [33]	18	Reading aloud > object naming	8
Rapp and Lipka [34]	10	Words > fixation	9
Rumsey et al. [35]	14	Phonological > fixation	15
Seghier et al. [36]	43	Reading aloud > fixation	10
Tan et al. [37]	10	Reading aloud regular words > baseline	37
Tan et al. [38]	6	Homophone judgment > fixation	38
Vigneau et al. [39]	23	Word reading and word listening > rest	14
Xiao et al. [40]	14	Real word lexical decision > rest	21
Xu et al. [41]	12	Word rhyming > baseline	9

*N* number of subjects

To find the co-activation relationships of the brain regions from the above dataset, we first extracted the activation information and converted it into a text file that included the peak coordinates (x, y, z). All of the coordinates were transferred to the corresponding MNI coordinates. Next, using formula (3), the brain coordinates were mapped to the corresponding brain regions in which the probability-activated threshold was defined as 0.9. Next, the A-Close algorithm was used to calculate the associated network of related brain regions, among them, the A-Close algorithm of minimum support was defined as 0.8 and the minimum confidence as 0.7. Finally, we used Pajek [42] to graph the experimental results (Pajek can be applied to analyze large-scale complex networks and is a powerful tool for studying a variety of complicated nonlinear networks).

## Results and discussion

**Fig. 1:**
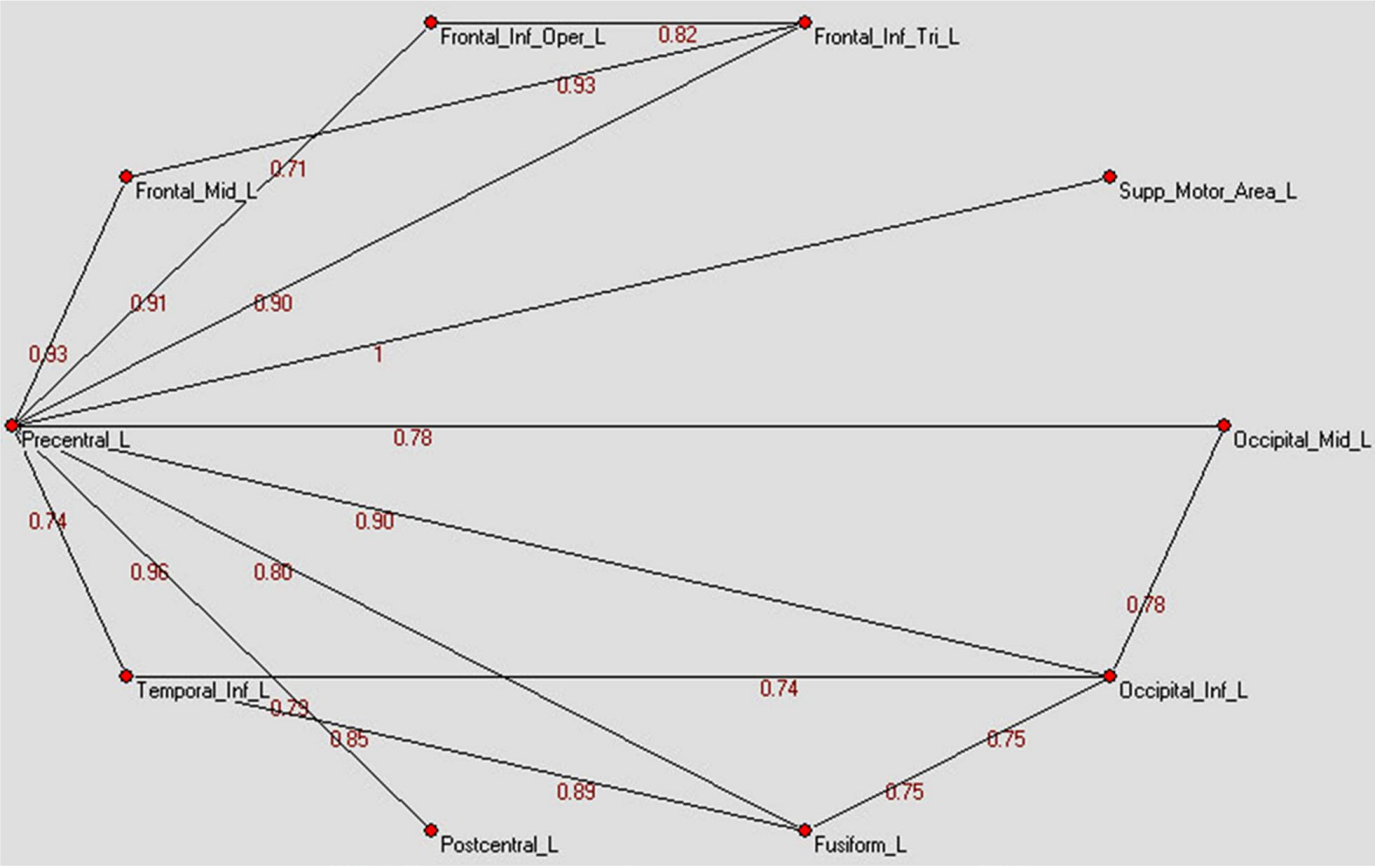
The co-activation relationships related to reading words

Our meta-analysis method examined how orthography is mapped to the functional connections related to phonology. The experimental results are shown in Fig. 1, wherein the weight value in red represents the confidence coefficient of each edge.

The weight values (red numbers) indicate the confidence coefficient of each edge (Frontal_Inf_Oper_L: left inferior frontal gyrus, opercular part; Frontal_Inf_Tri_L: left inferior frontal gyrus, triangular part; Frontal_Mid_L: left middle frontal gyrus; Fusiform_L: left fusiform gyrus; Occipital_Inf_L: left inferior occipital gyrus; Occipital_Mid_L: left middle occipital gyrus; Postcentral_L: left postcentral gyrus; Precentral_L: left precentral gyrus; Supp_Motor_Area_L: left supplementary motor area; Temporal_Inf_L: left inferior temporal gyrus).

As shown in Fig. 1, the left precentral gyrus, left fusiform, and left occipital lobe are connected, while the left temporal gyrus and fusiform gyrus are connected to the left precentral gyrus. There is one edge connection line between the left precentral gyrus and left inferior frontal gyrus. There is also one edge between the left middle frontal gyrus and left precentral gyrus and one between the left supplementary motor area and left precentral gyrus.

The left precentral gyrus is the hub for all of the connections, and the areas that are most frequently co-activated along with the left precentral gyrus are the left frontal lobe, left postcentral gyrus, left inferior occipital, and left supplementary motor area.

The results showed that the network extends throughout the frontal and occipital areas of the brain. The posterior network runs from the occipital lobe to the precentral gyrus and through the fusiform gyrus, superior temporal gyrus, and middle occipital gyrus, among others. The more anterior network includes the middle frontal gyrus, inferior frontal gyrus, and supplementary motor area. The entire network terminates at the precentral gyrus.

The occipital area in cognitive science is referred to as the visual cortex, and its main function is to process visual information [43]. Because reading involves transforming visual text into information that can be encoded in the brain, the functional network for reading likely involves the occipital area. Orthographic analysis is often performed in the fusiform gyrus region [44, 45]; in the present study, one edge existed between the left inferior occipital gyrus and left inferior temporal gyrus (Fig. 1), which is consistent with the results reported by Price [7]. The left inferior temporal gyrus is thought to be a memory storage area [46]. Therefore, after the orthographic analysis stage is complete, the fusiform is likely involved in the orthographic coding stage, while phonology processing occurs in the brain’s memory area. Finally, the circuit is completed in the posterior region of the brain.

Two pathways are activated simultaneously during reading [47, 48], namely the phonetic and semantic pathways. The frontal lobe is mainly involved in grammar processing, semantic analysis [49], and word generation; thus, the network in the anterior part of the brain consists of the entire inferior frontal gyrus (opercular, orbital, and triangular portions). Another part of the network, the middle frontal gyrus, mainly executes semantic retrieval [50]. Both silent reading and reading aloud require the precentral gyrus and posterior central gyrus, which are involved in speech output [51, 52]; therefore, the precentral gyrus may be the hub node and output node of the network.

Collectively, the co-activation relationships of the brain regions (regarding how orthography is mapped to phonology when performing reading tasks) obtained with our analysis method were in accordance with the network reported in the literature review by Price [7]. The main difference between our study and the review is that in addition to containing the brain networks derived from our meta-analysis of functional connections reported in the literature, the review also contains a dorsal pathway that includes the dorsal precentral gyrus and extends from the superior temporal gyrus to the inferior occipital lobe. However, in the summary by Price [7], it is unclear whether the dorsal pathway exists. Our meta-analysis method indicates that the dorsal pathway likely does not exist. The reason for this belief is based on the following principles of the association rule algorithm: (1) more than 20 % of the articles did not simultaneously refer to the mentioned brain regions in the dorsal pathway, and (2) if more than 80 % of the articles referred to the mentioned brain regions in the dorsal pathway, then the confidence coefficient among the brain regions in the dorsal pathway would be lower than 70 %. However, this remains to be confirmed.

Furthermore, the results of the connectivity analysis can be used as a priori knowledge for a DCM analysis [53, 54]; this would produce a significant dimension-reducing effect when performing the DCM analysis, thus increasing the efficiency of the analysis. In this study, some brain areas had no connections (such as the left inferior occipital gyrus and left middle frontal gyrus), which may not need to be considered in the corresponding DCM analysis. Therefore, when performing DCM analyses for multiple ROIs, the reduction in the calculation of a single edge will reduce the dimension in an exponential order, thus significantly increasing the DCM analysis efficiency. The related comparative validation will be performed in a future study.

The meta-analysis method presented herein can determine the co-activation relationships among the brain regions activated in studies utilizing similar tasks; however, our study also has some limitations. First, we selected a meta-analysis dataset from a review that may have included the author’s subjective views; in the future, we will apply this method to a literature database. Second, in this paper, we used the AAL atlas to define the brain regions. As more research on the brain is conducted, atlases that are more precise may become available. The use of such precise atlases will improve the accuracy of the experimental results.

## Conclusions

This article proposes a new meta-analysis method to find brain network connection models. This method employs data mining to discover the co-activation relationships among brain regions based on activation data reported in the relevant literature. Furthermore, studies utilizing word-reading tasks reported in a review by Price [7] were analyzed and the feasibility and robustness of this method were verified. Our meta-analysis method may enable us to better understand the brain’s processing patterns during certain cognitive tasks. In particular, the results of the connectivity analysis can provide reliable a priori knowledge for future experimental research. For example, the results of the connectivity analysis can be used in corresponding DCM analyses to eliminate edges that do not need to be considered in order to increase the DCM calculation efficiency.

## Abbreviations

AAL: automatic anatomical label
ALE: activation likelihood estimation
DCM: dynamic causal modeling
fMRI: functional magnetic resonance imaging
MACM: meta-analytic connectivity modeling
MNI: Montreal Neurological Institute
ROIs: regions of interest
